# Isolation and characterization of multiple-stress tolerant bacteria from radon springs

**DOI:** 10.1371/journal.pone.0299532

**Published:** 2024-03-07

**Authors:** Elizaveta Timkina, Marketa Kulišová, Andrea Palyzová, Helena Marešová, Olga Maťátková, Tomáš Řezanka, Irena Jarošová Kolouchová

**Affiliations:** 1 Department of Biotechnology, University of Chemistry and Technology, Prague, Czech Republic; 2 Institute of Microbiology, Czech Academy of Sciences, Prague, Czech Republic; Alagappa University, VIET NAM

## Abstract

Radon springs, characterized by their high concentrations of radon gas (Rn^222^), are extreme environments with unique physicochemical conditions distinct from conventional aquatic ecosystems. Our research aimed to investigate microbial life in radon springs, focusing on isolating extremophilic bacteria and assessing their resistance to adverse conditions. Our study revealed the prevalence of Actinomycetia species in the radon spring environment. We conducted various tests to evaluate the resistance of these isolates to oxidative stress, irradiation, desiccation, and metal ion content. These extremophilic bacteria showed overall higher resistance to these stresses compared to control strains. Lipidomic analysis was also employed to provide insights into the adaptive mechanisms of these bacteria which were found mainly in the correlations among individual clusters and changes in content of fatty acids (FA) as well as differences between content and type of FAs of environmental isolates and type strains.

## 1 Introduction

The number of prokaryotes in the waters of deep geological rifts and layers is reported to be 10^3^−10^5^ microbial cells per millilitre [1]. Chemolithotrophic organisms are primarily found in those environments [2], especially in groundwater [3, 4]. Biotopes of hyperalkaline [5–8], low-temperature [9] and high-temperature spring waters [10, 11] have been widely investigated. Waters occurring in deep geological layers are of particular interest due to the possible discovery of new isolates with the potential to produce new biologically active molecules [12]. The geological origin of microbial isolates seems to fundamentally influence their properties, even among seemingly genetically identical microorganisms [13]. A special type of groundwater environment is water containing radioactive isotopes, especially ^226^Ra and ^222^Rn produced by radioactive decay, found in a limited number of places around the world with different radon activity [13–16]. Radon is a radioactive gaseous element emitting alpha particles and thereby generating free radicals, whose interactions with biological molecules cause peroxidation of cellular lipids and DNA damage [4]. So far, radon waters have mostly been monitored from the point of view of chemical composition [17, 18] or in spite of the influence of radon content on the properties of drinking or spa water [19–22]. The microbial ecology of waters containing radioactive isotopes however remains mostly unexplored.

Research conducted on radon occurrence in water sources within the Czech Republic has demonstrated that the principal concentration of radioactive sources is predominantly associated with geological formations in close proximity to the Lugian orthogneiss, Tanvald, and Krkonoše (Giant Mountains) granite formations. The water in these areas is characterized by low mineralization (below 200 mg/L), along with elevated levels of uranium. Additionally, there is a prevalence of manganese over iron, temperatures in the range of 5 and 12°C, an acidic pH, and a season-dependent high oxidation-reduction potential [23]. Some studies demonstrate that the ability of microorganisms to accumulate manganese leads to their higher resistance to stress conditions and helps in the catalytic detoxification of ROS [24]. The town of Jáchymov, located in the western part of the Czech Republic, is renowned for its radon-rich springs. The Svornost mine, the world’s first and for a long time the only radium mine, is also the deepest one devoted to the extraction of water and not raw materials. The waters are radon-saturated due to radium decay in Jáchymov subsoil and are used for their therapeutic benefits connected with their chemical properties. This water, which is approximately thirteen thousand years old, is used as spa water under the medical supervision for the treatment of patients with nervous and rheumatic disorders. Radon activity in Jáchymov significantly surpasses the levels found in the aforementioned springs, ranging from 5 to 20 kBq/L [25].

There are not many scientific publications on the microbial biodiversity of spring or sediment systems in extreme environment focusing on linking the properties of the extreme environment with the mechanisms of adaptation of microorganisms to the conditions. Millan et al. [26] focused on diatom communities in La Montagne, Châteldon, France; Weidler et al. [15] studied morphological diversity in biofilms in underground thermal alpine springs. Life in these extreme systems requires many adaptations of the microorganism—modifications in the composition of proteins, DNA and cell membranes and intracellular accumulation of low molecular weight compounds [27–30]. It is interesting to note that among extremophilic microorganisms, so far, the most attention has been paid to the thermophiles. Their mechanisms of adaptation are studied in detail, especially their enzymes [31], which are used in industrial and biotechnological applications [32]. For this reason, it is desirable to extend this knowledge to the environment of radon mineral waters. The most studied radioresistant extremophile is *Deinococcus radiodurans*, which has become a model microorganism for radiation resistance, oxidative stress resistance and repair mechanisms as well as potential target microorganism for bioremediation, biomedical and biocatalysis applications [33].

In recent years, several culture-independent studies have focused on underground aquifers and the microbial communities, providing a novel perspective on the metabolic pathways and biodiversity of such habitats [34, 35]. While culture-dependent and culture-independent studies often appear to focus on different bacterial populations, a model study demonstrated that the dominant phyla in the environment remained consistent regardless of whether a culture-based or culture-independent approach was employed. Despite the initial promise of high-throughput sequencing (HTS) techniques potentially supplanting bacterial culturing [36], cultivation methods continue to play a crucial role. In many cases cultivation methods are essential for enhancing taxonomic accuracy and expanding diversity coverage [37]. Furthermore, cultivation techniques remain indispensable for retrieving complete genomes and facilitating physiological and metabolic investigations, as demonstrated by research [38].

A growing number of newly identified Actinomycetia species are consistently being documented in natural environments, particularly in extreme biomes characterized by extreme environmental conditions. These include systems with exceedingly high or low temperatures, nutrient scarcity, minimal organic carbon availability, elevated levels of oxidative compounds, high level of UV radiation, extreme pH or salinity, toxic metal concentrations, and various other abiotic stressors [37, 39, 40]. These findings provide valuable insights into Actinomycetia species unique adaptations and contributions to ecosystem dynamics. They play pivotal roles in nutrient cycling, organic matter decomposition, contributing to the overall sustainability of these harsh ecosystems [14, 15]. Moreover, their ability to produce bioactive secondary metabolites helps protect them from environmental stresses and plays a crucial role in shaping microbial communities [41]. Several Actinomycetia species were studied regarding their resistance to oxidative stress. Actinobacteria are known for their relatively high resistance to gamma and ultraviolet radiation, which is why they are also found in places with significant radioactivity. For example, three species from the class Rubrobacteria have been identified as radiotolerant: *Rubrobacter radiotolerans*, *R*. *xylanophilus*, and *R*. *taiwanensis* [42–44]. The genus *Kineococcus* also comprises species known for their remarkable resilience to ionizing radiation and extreme desiccation. Notably, the species *K*. *radiotolerans*, isolated from a sample obtained from a radioactive waste container, exhibits exceptional resistance. Exposure to a gamma radiation dose of 3 kGy leads to a reduction in viable cell count by an order of magnitude [45].

When exposed to environmental stress such as changes in temperature, pH, osmotic pressure radiation, and oxidative conditions, bacteria are able to modify the composition of fatty acids (FAs) in their membrane. Bacterial cytoplasmic membranes can compensate for modified growth conditions by a process known as homeoviscous adaptation, which adapts the membrane so that it can maintain fluidity when the environment changes [46]. UV exposure was also documented to cause FA membrane modifications [47]. There are several enzymes, known as FA desaturases which have been proposed for this modification. These enzymes have also been shown to not be produced under normal conditions, but induced by environmental stress [48].

The investigation of microbial life in radon springs carries numerous biotechnological implications. For instance, the enzymes and metabolic pathways evolved by radon spring bacteria might be used in various industrial processes, including bioremediation of radionuclide-contaminated sites and the development of bioengineered systems capable of withstanding harsh conditions [41].

In this regard, a culture-based approach was selected for the presented study. The resulting bacterial library revealed the prevalence of Actinomycetia species. Further tests were conducted to assess the rate of resistance to oxidative stress conditions and possible adaptation in which bacteria isolated from underground radon springs under various physicochemical stresses showed overall higher resistance to these stresses than the control strains.

In summary, our research efforts encompassed the isolation and taxonomic classification of extremophilic bacteria, the assessment of the impact of stress factors, including radiation, desiccation, and metal ions content, on the biochemical properties of strains and their comparative analysis. The lipidomic characterization of these strains was also included as a complementary factor to the overall characterization of possible adaptative mechanisms that were found.

## 2 Methods

### 2.1 Bacterial strains and culture conditions

Isolated strains from Jáchymov springs were grown in half concentrated TSB broth and TSA agar. *Deinococcus radiodurans* R1 (= CCM 1700^T^ = DSM 20539), grown in B8 broth, and *Escherichia coli* CCM 4517 (= DSM 1116), grown in TSB broth, were used as positive and negative control strains, respectively. All bacterial isolates from Jáchymov springs and *D*. *radiodurans* R1 were incubated at 30°C under aerobic conditions for 72 hours. *E*. *coli* CCM 4517 was grown at 37°C for 24 hours. At the end of cultivation harvested cells were washed twice with sterile physiological saline solution and bacterial suspension of optical density (measured at 600 nm) 0.5 was prepared (equal to 10^7^−10^8^ of viable cells) for further tests.

### 2.2 Sample collection

We confirm that the authorities of Svornost mine have provided formal authorization for the collection of water from the underground spring within the mine premises. The Svornost mine is accessible for scientific purposes, access was approved by the mine director, Ing. Jiří Pihera. Water samples were aseptically collected in the presence of mine director from four underground radon springs (Agricola, Běhounek, C1 and Curie) in Jáchymov, Czech Republic. Sterile conical tubes containing sterile LB medium or YPD medium were used for sampling. Radon water was added to the tubes to achieve a tenfold dilution of the initial concentration of nutrients in these media. The samples were further cultured at a temperature of 30°C (samples from the Agricola, C1 and Curie springs) or 37°C (samples from the Běhounek spring).

After 30 days of culture in conical tubes, each enriched sample was diluted to achieve a cell concentration of 1×10^3^ mL^-1^ and inoculated onto solid media. Solid media used: Reasoner’s 2A (R2A) agar; noble agar (1.8%) and lactate (0.5%) medium; noble agar (1.8%). Samples from individual springs were cultured on media prepared from the corresponding radon water samples. Petri dishes were cultured at 30°C (Agricola, C1 and Curie) or 37°C (Běhounek) for 1–3 weeks. Several colonies with different morphologies were transferred to Petri dishes with TSA agar and cultured at 30°C or 37°C for 24 h.

### 2.3 Molecular identification of isolates

Chromosomal DNA was isolated using a commercial High Pure PCR Template Preparation Kit (Roche) according to the manual. The 16S rRNA gene region was amplified using PCR HS Taq Mix (PCR Biosystems) and universal bacterial PCR primers (27F + 1492R or 359F + 1492R) in the PCR reaction: 95°C- 4 min; 35 cycles of 95°C- 30 s, 55°C- 30 s, 72°C- 90 s; 10 min -72°C. Purification of PCR amplicons was performed using the High Pure PCR Product Purification Kit (Roche, Switzerland) according to the manufacturer’s protocol. PCR amplicon sequencing was performed on an ABI PRISM 3130xl genetic analyzer (Applied Biosystems, USA). ChromasLite software (Technelysium Pty Ltd., Australia) was used to edit the sequences, which were then assembled using Lasergene software (DNASTAR, Inc., USA). Sequence similarities of the 16S rRNA gene were searched in the GenBank data library using the BLASTN program (NCBI, USA). Sequences were aligned using the Multiple Sequence Comparison by Log Expectation algorithm (MUSCLE) [49] and the Maximum Composite Likelihood model integrated in the MEGAX program was used to infer evolutionary history [50].

### 2.4 Resistance to ionizing radiation

To test the resistance to UV-C irradiation, bacterial suspension (4 ml) was pipetted into an empty sterile Petri dish. The suspension was irradiated in an open Petri dish with UV-C radiation from 40 cm using an OSRAM PURITEC HNS 30W G13 UV lamp with the highest intensity at a wavelength of 254 nm. Samples were taken from the dish at times 2.5 (1.2 kJ/m^2^); 5 (2.3 kJ/m^2^); 7.5 (3.5 kJ/m^2^); 10 (4.7 kJ/m^2^); 15 (7 kJ/m^2^) and 30 (14.0 kJ/m^2^) minutes. A non-irradiated bacterial suspension served as a control [51, 52].

To test resistance to gamma irradiation the samples (1 ml of bacterial suspension in a microtube) were irradiated with a circular electron accelerator microtron MT25. This source of gamma irradiation has a continuous energy spectrum with a maximum energy of 16.5 MeV. Total of 5 doses of gamma radiation were tested—0.25 kGy, 0.5 kGy, 1 kGy, 1.5 kGy and 2 kGy. The absorbed dose was measured with a calibrated ionization chamber TN34045 (PTW Freiburg, Germany) and a Keithley 617 electrometer (Keithley, USA). A non-irradiated bacterial suspension served as a control. To assess the number of viable bacterial cells, serially tenfold diluted samples were spread on the respective agar plates and incubated for 48 to 72 h at 30 or 37°C. The experiments were performed in three parallels and three independent repetitions.

### 2.5 Resistance to oxidative stress

The bacterial cells (optical density at 600 nm 0.5) were either exposed to hydrogen peroxide for 10 minutes with final concentration in the micro tube 0.26, 0.44, 0.88, 1.76 and 2.65 mol/L, or to mitomycin C for 30 minutes with final concentration in the micro tube 5, 10 and 15 μg/mL as was previously described [51]. Then cell suspension was centrifuged in microtubes, and the pellet was washed twice with physiological saline solution and resuspended. 30 μL of each of the above-described bacterial culture treated with hydrogen peroxide or mitomycin C and 270 μL of the appropriate culture medium were pipetted into the wells of a microtiter plate. The plate was cultured for 72 hours in the Bioscreen C culture device (Finland), the growth was determined spectrophotometrically in the range of wavelengths 420 – 580 nm. The results were expressed as a percentage of the achieved optical density relative to the untreated culture. The experiments were performed in three parallels and three independent repetitions.

### 2.6 Resistance to desiccation

Cell suspension was pipetted into the microtiter plate and left to dry in. The plate was placed in a desiccator and left at a temperature of 25°C. Cell viability was measured after 14 and 28 days using the fluorescent dyes propidium iodide (PI) and SYTO 9 (after rehydration of the cells with PBS solution for 1 hour) as was previously described [53]. A bacterial suspension that was not subjected to the desiccation served as a control. After rehydration, 200 μL of the bacterial suspension was taken from the well into a microtube, and 2 μL of SYTO 9 solution (0.1 mmol/L) and 2 μL of PI solution (1 g/L) were added. The tube was mixed and left for 5 to 10 minutes in the dark. Stained bacterial cells were analysed using a FACS Aria III flow cytometer (USA) and data were processed using FACS Diva 8.0 software. Excitation was induced by a blue argon laser with a wavelength of 488 nm. In the case of the dye SYTO 9, the emission was captured by a filter with parameters 530/30, in the case of the dye PI, a filter with parameters 575/26 was used [53]. The obtained results were evaluated as the percentage of living cells after 14 and 28 days of drying using the procedure previously described [54].

### 2.7 Determination of the intracellular concentrations of Mn: Fe

Intracellular concentrations of manganese and iron were determined using inductively coupled plasma mass spectrometry (ICP-MS) as described in Daly et al., 2004 [55]. The cells were subsequently lyophilized. 2 mL of concentrated HNO_3_ was added to the cells for analysis. The samples were placed in a water bath at 80°C for 1 hour. The treated samples were diluted with deionized water so that the final concentration of HNO3 was 2%. The cells were washed with PBS and EDTA solution, and after adding indium internal standard solution the Fe and Mn content was analyzed by NexION 350D mass spectrometer (Perkin Elmer, Canada). The monitored nuclides were the analytes ^55^Mn and ^57^Fe, and the internal standard ^115^In. The following conditions were set for the measurement: plasma input 1100 W, fog chamber temperature 4°C, Ar flow through the nebulizer 0.76 L/min, Ar plasma flow 11 L/min, Ar auxiliary flow 1 L/min, total integration time 15 seconds.

### 2.8 Isolation of lipids and fatty acid methyl ester (FAME) characterization

The extraction procedure was based on the method described by Bligh and Dyer [56]. Briefly, the lyophilized cells were suspended in a dichloromethane:methanol mixture (2:1, v/v) for 30 minutes with stirring, after which dichloromethane and water were added, and the dichloromethane phase was evaporated to dryness under reduced pressure. Aliquot part (1 mg) of total lipids was dissolved in 1 mL diethyl ether and 3 mL methyl acetate. 25 μL of 1 M sodium methoxide in methanol was added. The sample was incubated for 50 min at room temperature. A saturated oxalic acid solution (0.5 mL) was added, with brief agitation, to neutralize the solution. The solvent was removed under nitrogen, and an appropriate volume of hexane was added to bring the sample to the concentration required for analysis.

GC-MS of fatty acid methyl ester (FAME) mixture was done on a Finnigan 1020 B (Finnigan MAT, Bremen, Germany) in electron impact (EI) mode. Splitless injection was at 100°C, and a fused silica capillary column (Supelcowax 10; 60 m x 0.25 mm i.d., 0.25 mm film thickness; Supelco, Prague) was used. The temperature program was as follows: 100°C for 1 min, subsequently increasing at 20°C/min to 180°C and at 2°C/min to 280°C, which was maintained for 1 min. The carrier gas was helium at a linear velocity of 60 cm/s. All spectra were scanned within the range of m/z 50–500. FAMEs were identified according to their mass spectra [57, 58] and using a mixture of chemical standards obtained from Merck (formerly Sigma Aldrich, Prague, Czech Republic).

### 2.9 Statistical analysis

Statistical analysis was performed in RStudio, where one-way Analysis of Variance (ANOVA) with Tukey post hoc test and Correlation Analysis was used for the comparison and evaluation of the isolate parameters. In the case of non-normal distributions, the data were transformed to obtain the normal distributions, using natural logarithm before statistical analysis. Dixon’s Q test was used for the detection of outliers in data obtained (the determination of each parameter was performed in five parallels, the deviation of the five determinations was less than 5%). The analysis of fatty acids composition and carbon source utilization was performed in three parallels. The standard deviation of the measurement was less than 5%.

## 3 Results and discussion

### 3.1 Identification of isolates

Isolates were obtained from four Jáchymov radon springs (Agricola, Curie, Běhounek, and C1). Radon spring locations and their physicochemical characteristics are shown in Table 1.

**Table 1:** Physicochemical characteristics and geographic coordinates of the Jáchymov radon springs (Czech Republic), sources of studied isolates (mean values during one year).

	Agricola	Curie	Běhounek	C1
Q flow (l/min)	5.1	26.4	273.8	21.8
T temperature (°C)	27.6±0.2	28.6±0.1	37.4±0.1	29.7±0.1
Rn (kBq/l)	23.42	5.21	8.09	9.98
pH	7.1	7.4	7.1	7.2
Conductivity (at 25°C; mS/cm)	0.613	0.658	0.641	0.564
ORPH (mV)	215	230	380	200
Total mineralization (mmol/l)	13.7	14.3	14.3	13.8
Geographic coordinates	X: 995 847,29	X: 995 881,00	X: 996 280,00	X: 995 846,58
	Y: 844 742,74	Y: 844 712,00	Y: 844 510,00	Y: 844 743,37
	Z: 259,3	Z: 235,7	Z: 263,7	Z: 259,3

**Fig 1:**
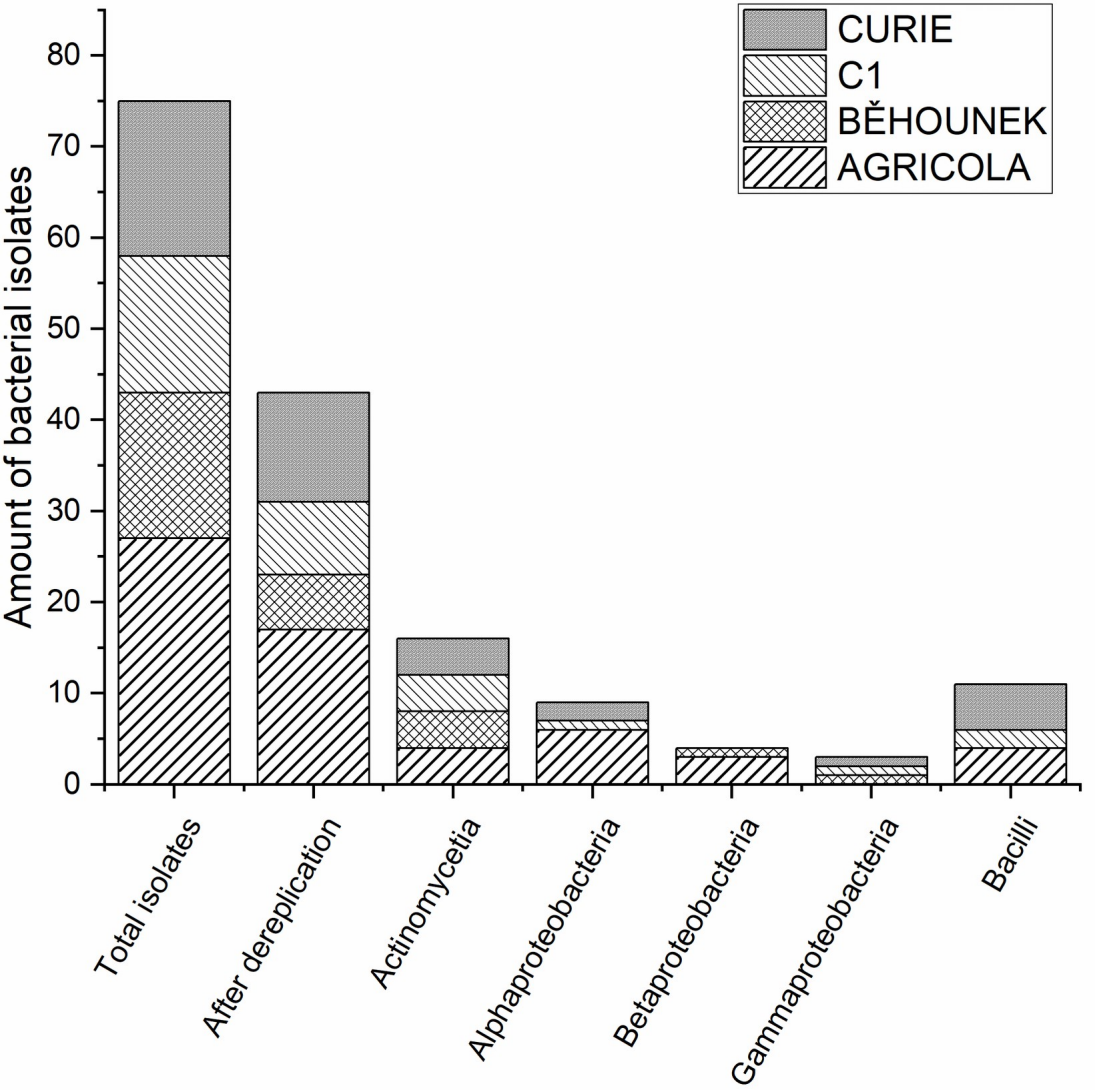
Determination of cultivation yields and proportion of isolated bacteria groups (based on 16S rRNA analysis) at the class level for each spring studied.

Each spring appears to harbour a distinct microbial community, as evidenced by the varied distribution of bacterial classes among the isolates [13]. The enrichment protocol and isolation procedure used in this study resulted in obtaining 75 pure cultures spanning 5 phylogenetic classes (Fig 1). The highest number of isolates was obtained from the Agricola spring, followed by Curie, Běhounek and C1 spring. All studied isolates were successfully transferred to commonly used bacteria culture media, proved to be suitable for cryo conservation with high survival rate. Total of 16 bacterial isolates present in all studied springs were identified to belong to Actinomycetia class based on 16S rRNA with the similarity level above 99%. Actinomycetia are frequently isolated from extreme environments such as hot springs (e.g. *Dietzia* sp. MG4 strain isolated from the Sirch Hot Spring, Iran [59]), radioactive sites (e.g. *Kineococcus radiodurans* isolated from radioactive waste [45]), deserts (*Geodermatophilus* sp. isolated from Sahara Desert [60]). Presence of Actinomycetia in radon rich environments was previously confirmed in culturomic studies of radon springs in Japan [61] and Iran [62] and by culture independent studies conducted in Australia [14] and Austria [15]. The study of the prevalence of Actinomycetia species, including the actinomycete genus *Kocuria*, in these radon underground springs is vital for a nuanced understanding of their ecological roles. Actinomycetia, a diverse bacterial group, demonstrates unique adaptations to extreme environments, as evidenced by their presence in radon-rich springs. Within this context, the genus *Kocuria*, a member of Actinomycetia, stands out for its known ability to produce bioactive compounds with antimicrobial properties which may significantly impact the presence of other microorganism. The ecological implications therefore extend beyond the mere presence of Actinomycetia to their potential influence on microbial community structures, contributions to nutrient cycling, and impacts on overall biodiversity within these ecosystems. Further studies are necessary to unveil the intricate ecological dynamics at play in radon underground springs, providing valuable insights into the broader ecological consequences associated with the prevalence of Actinomycetia.

Another numerous group of microorganisms found in the radon springs belonged to the class Bacilli (11 isolates), followed by Alphaproteobacteria (9 isolates), Betaproteobacteria (4 isolates) and lastly Gammaproteobacteria (3 isolates). In the spring Běhounek no bacteria identified as Alphaproteobacteria nor Bacilli were detected. Gammaproteobacteria were absent from the Agricola spring and Betaproteobacteria were absent in both C1 and Curie springs.

**Fig 2:**
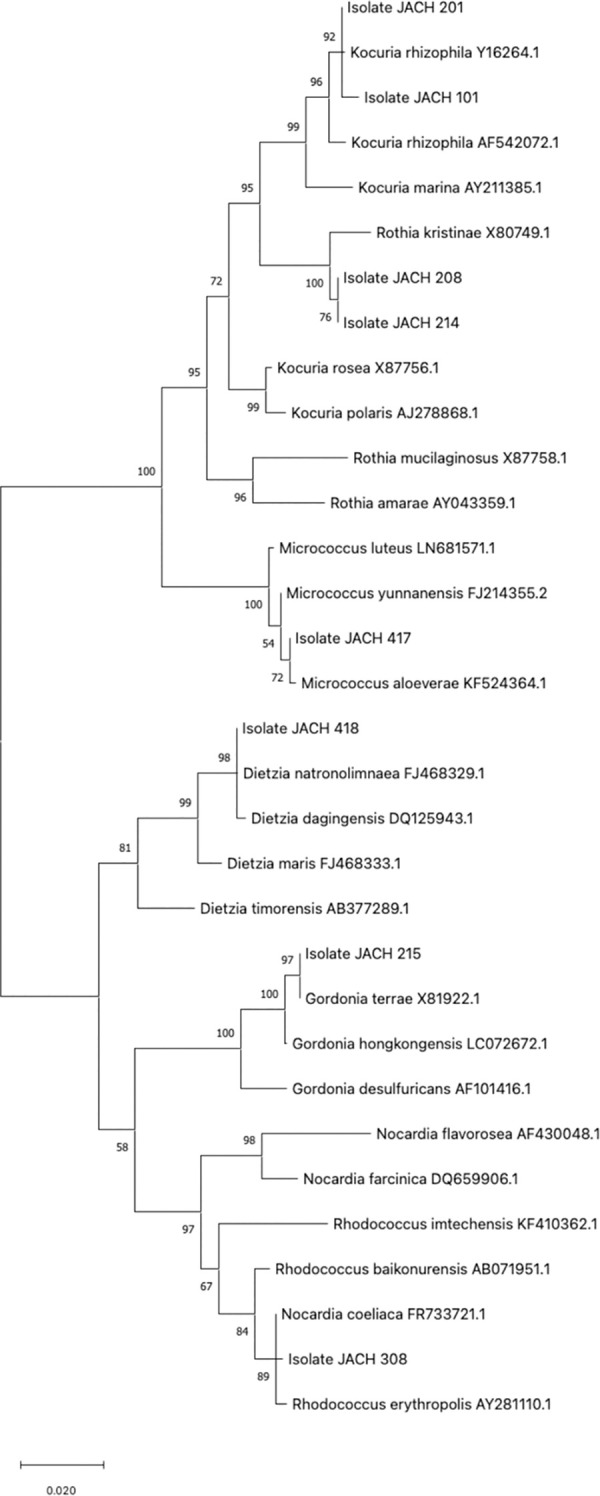
Phylogenetic tree constructed for the studied bacterial isolates based on 16S rRNA alignment. The evolutionary history was inferred by using the Maximum Likelihood method and Tamura-Nei model. The tree with the highest log likelihood (-4078.15) is shown. The percentage of trees in which the associated taxa clustered together is shown next to the branches. Initial tree(s) for the heuristic search were obtained automatically by applying Neighbor-Join and BioNJ algorithms to a matrix of pairwise distances estimated using the Tamura-Nei model, and then selecting the topology with superior log likelihood value. The tree is drawn to scale, with branch lengths measured in the number of substitutions per site.

Eight isolates belonging to Actinomycetia (Table 2), which differed in the screening by the degree of resistance and phylogenetic proximity, were further tested for their resistance to different types of stress. Phylogenetic analysis of the isolates revealed the formation of two distinct groups (Fig 2): i) a cluster closely related to *Gordonia*, *Dietzia*, *Rhodococcus*, and *Nocardia* species (isolates 308, 215, and 418) and ii) a separate cluster with *Kocuria*, *Rothia*, and *Micrococcus* species (isolates 101, 201, 208, 214, and 417). In addition, the phenotype of all isolates was characterized, and their biochemical and morphological traits are shown in S1 Table. The enzymatic reactions for the bacteria were tested using the API ZYM test system (BioMérieux SA, France) following the manufacturer’s instructions. Genus *Gordonia* and *Nocardia* were originally enclosed in the “rhodochrous complex” due to similar morphological traits [63], but later studies showed biochemical and genetical difference between them. Heterogeneity within the genus *Rhodococcus* was firstly detected in the studies of mycolic acid (high-molecular-weight α-branched 3-hydroxy fatty acid) and menaquinone composition and later confirmed by 16S rRNA analysis [64, 65]. Genus *Dietzia* was firstly described in 1995 after phylogenetic analysis of several *Rhodococcus* sp. members [66].

**Table 2:** List of isolates selected for the resistance study.

Spring, activity of radon	Isolate	Identification 16S rRNA (database GenBank NCBI)	Genetic code similarity	NCBI Accession number	Czech collection of microorganisms number
Agricola, 20 kBq/L	JACH 101	*Kocuria rhizophila*	99.29%	OR392465	CCM 9359
Běhounek, 10 kBq/L	JACH 201	*Kocuria rhizophila*	99.93%	OR742322	CCM 9360
	JACH 208	*Rothia kristinae*	99.82%	OR742339	CCM 9361
	JACH 214	*Rothia kristinae*	100%	OR742343	CCM 9362
	JACH 215	*Gordonia terrae*	99.79%	OR742344	CCM 9363
C1, 11 kBq/L	JACH 308	*Rhodococcus erythropolis / Nocardia coeliaca*	99.82% 99.82%	OR742402	CCM 9364
Curie, 5 kBq/L	JACH 417	*Micrococcus luteus*	99.82%	OR742403	CCM 9365
	JACH 418	*Dietzia natronolimnaea*	100%	OR742411	CCM 9366

*Kocuria* sp. was originally enclosed in genus *Micrococcus*, but based on polar lipids, fatty acids, menaquinone analysis and phylogenetic analysis a distinctive genus *Kocuria* was proclaimed [67]. A recent phylogenetic study shows that genus *Kocuria* is paraphyletic with respect to genus *Rothia* and *K*. *kristinae*, first isolated from human skin and described as *Micrococcus kristinae* [68] has more genetic similarity to *Rothia* compared to the remaining *Kocuria* species. Biochemical traits, lipid content and cell wall composition of *Kocuria* species appear to have no significant difference from *Rothia* sp. [69].

### 3.2 Ionizing radiation tolerance assay

**Fig 3:**
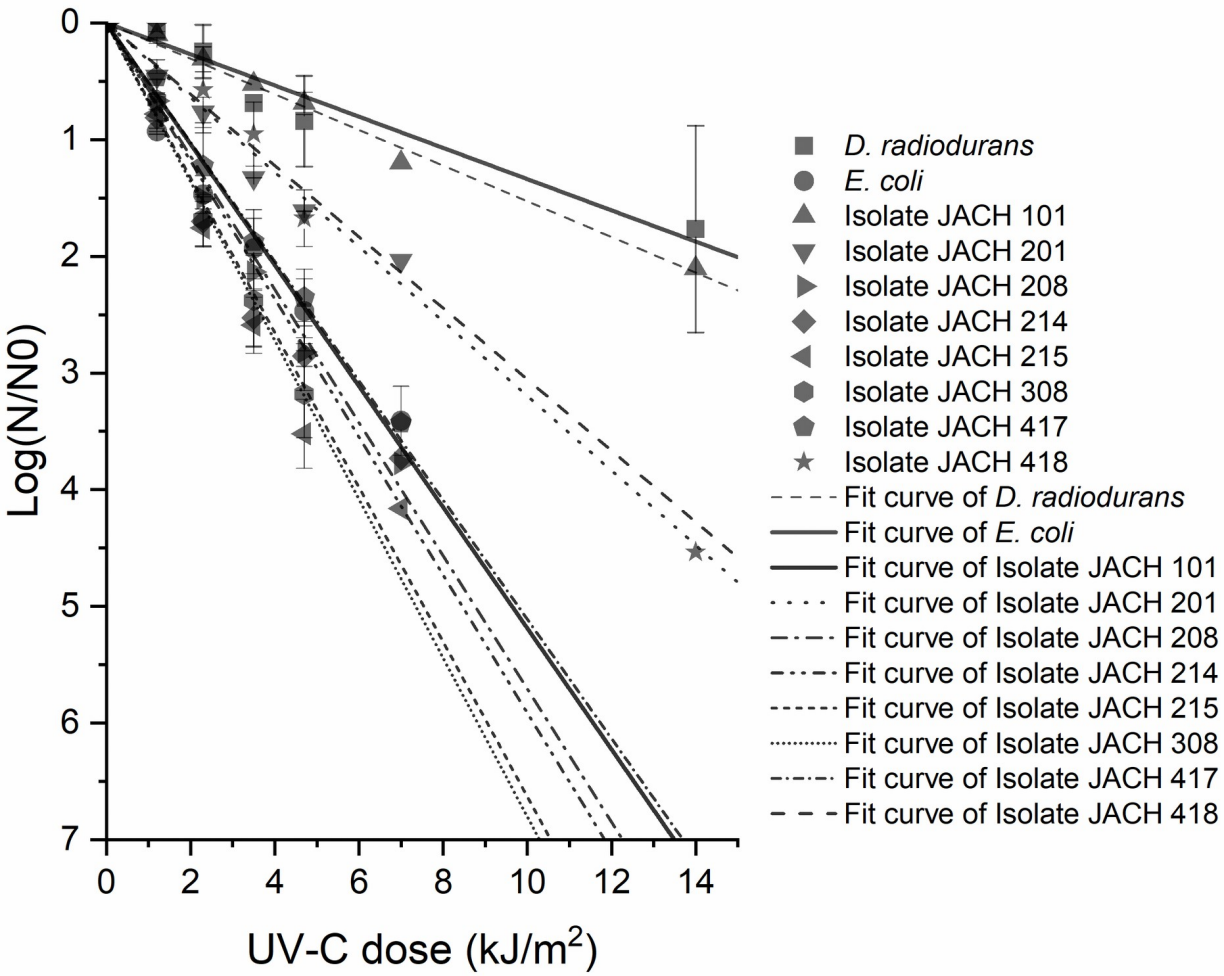
Survivability rates (−LogNN0_,_ N–CFU after exposure to a certain dose of UV-C, N_0_ –control CFU without exposure) of studied isolates and control strains (*D*. *radiodurans* R1 and *E*. *coli* CCM 4517) after exposure to UV-C irradiation.

The environmental isolates (101, 201, 208, 214, 215, 308, 417, 418) and control strains were exposed to six different doses of UV-C radiation (range 1.2–14.0 kJ/m^2^) to assess their level of resistance to UV-C. *D*. *radiodurans* R1 was used as a positive control strain and *E*. *coli* CCM 4517 was used as a negative control strain. The data acquired from these tests are depicted in Fig 3, where correlation between dose of irradiation and logarithm of survivability rate is expressed. The most resistant isolate 101 (*K*. *rhizophila*) showed high resistance to radiation, almost as high as the positive control *D*. *radiodurans* R1 which is widely used in literature as standard for high resistance evaluation [70]. After exposure to a dose of 1.2 kJ/m^2^, this isolate showed the highest cell viability compared to all other tested isolates from Jáchymov (80 ± 5% compared to the non-irradiated culture). Viable cells were detected after exposure up to a dose of 111.6 kJ/m^2^. Isolates 201 (*K*. *rhizophila*) and 418 (*D*. *natronolimnaea*) also demonstrated elevated resistance levels, in comparison with negative control strain *E*. *coli*. In contrast, all other isolates (isolate 208 –*R*. *kristinae*, 214—*R*. *kristinae*, 215 –*G*. *terrae*, 308 –*R*. *erythropolis/N*. *coeliaca*, 417 –*M*. *luteus*) exhibited either comparable or lower levels of UV-C resistance when compared against the negative control, *E*. *coli* CCM 4517.

Similar UV resistance has been documented in other representatives of *Kocuria* sp. (*K*. *rosea*, *K*. *erythromyxa*) or *Dietzia* sp., which were also isolated from radioactive sources [52, 59, 61, 62]. Notably, the UV resistance of *K*. *rosea* MG2 isolate from Ab-e-Siah spring in Iran was found comparable to that of *Deinococcus* sp. bacteria [52].

### 3.3 Gamma radiation exposure

**Fig 4:**
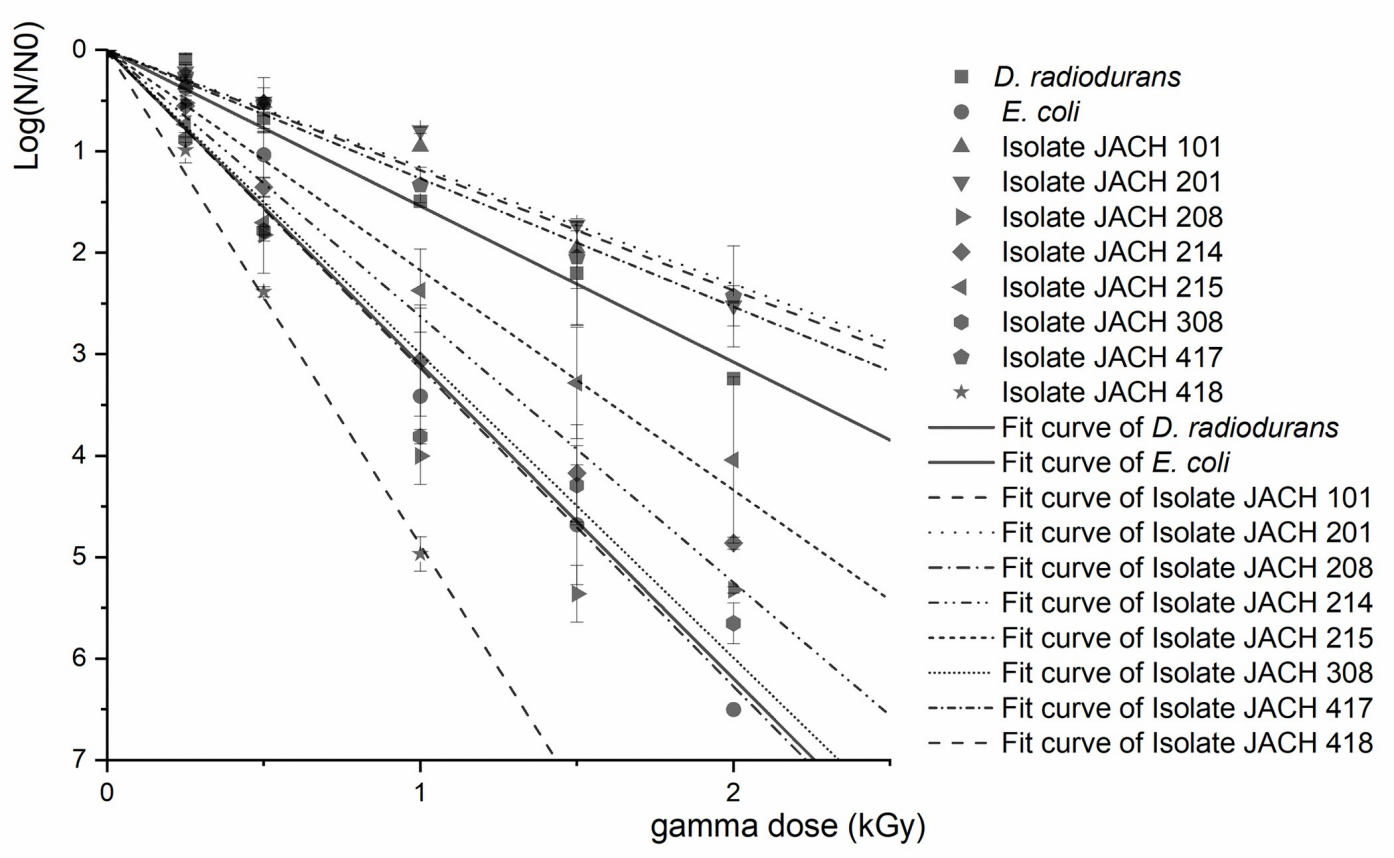
Survivability rates (−LogNN0_,_ N–CFU after exposure to a certain dose of gamma irradiation, N_0_ –control CFU without exposure) of studied isolates and control strains (*D*. *radiodurans* R1 and *E*. *coli* CCM 4517) after exposure to gamma irradiation.

When studying the ability of bacterial isolates to survive exposure to gamma radiation, five doses of radiation were tested: 0.25 kGy, 0.5 kGy, 1 kGy, 1.5 kGy, and 2 kGy. The obtained data are shown in Fig 4, depicting the relationship between the logarithm of the surviving proportion of bacteria and the dose of gamma radiation. The steeper the slope of the linear regression line representing each isolate in relation to the x-axis, the lower the resistance of the isolate to radiation. Isolates 201 (*K*. *rhizophila*), 417 (*M*. *luteus*), and 101 (*K*. *rhizophila*) demonstrated the highest resistance. Specifically, isolate 201 (*K*. *rhizophila*) showed a survival rate of 16 ± 2% compared to the unirradiated culture, and isolate 101 (*K*. *rhizophila*) exhibited 11 ± 5% live cells after irradiation with a dose of 1 kGy. Based on our findings, it can be concluded that isolates 101 (*K*. *rhizophila*) and 201 (*K*. *rhizophila*) display radioresistance. Following irradiation with a 1 kGy dose, the percentage of viable cells for both isolates remained above 10%—specifically, 10 ± 2% and 16 ± 1%. This underscores their ability to withstand the effects of radiation [71]. Isolate 417 (*M*. *luteus*) had a slightly lower survival rate at 5 ± 2%. All three isolates exhibited similar resistance to gamma radiation as the positive control culture of *D*. *radiodurans* R1 under the experimental conditions. Considering the increased presence of palmitoleic fatty acid in both environmental isolates and *D*. *radiodurans* R1, it is possible that the membrane composition and radioresistance could be connected. Several other isolates from Jáchymov springs (isolates 214—*R*. *kristinae* and 215 –*G*. *terrae*) displayed lower resistance in comparison to the significantly radioresistant isolates above, but even these showed higher resistance than that of the negative control, *E*. *coli* CCM 4517. The resistance to gamma radiation is often found in the genus *Kocuria* which was substantiated by studies, with Guesmi et al. (2021) reporting a D10 value of 2.9 kGy for *K*. *rhizophila* PT10 strain [72], and Asgarani et al. (2012) determining a D10 value of 2 kGy for *K*. *rosea* ASB-107 [62]. For the rest of isolates (isolate 208—*R*. *kristinae*, 308 –*R*. *erythropolis/N*. *coeliaca* and 418—*D*. *natronolimnaea*), gamma radiation had pronounced lethal effects.

### 3.4 Desiccation tolerance

**Fig 5:**
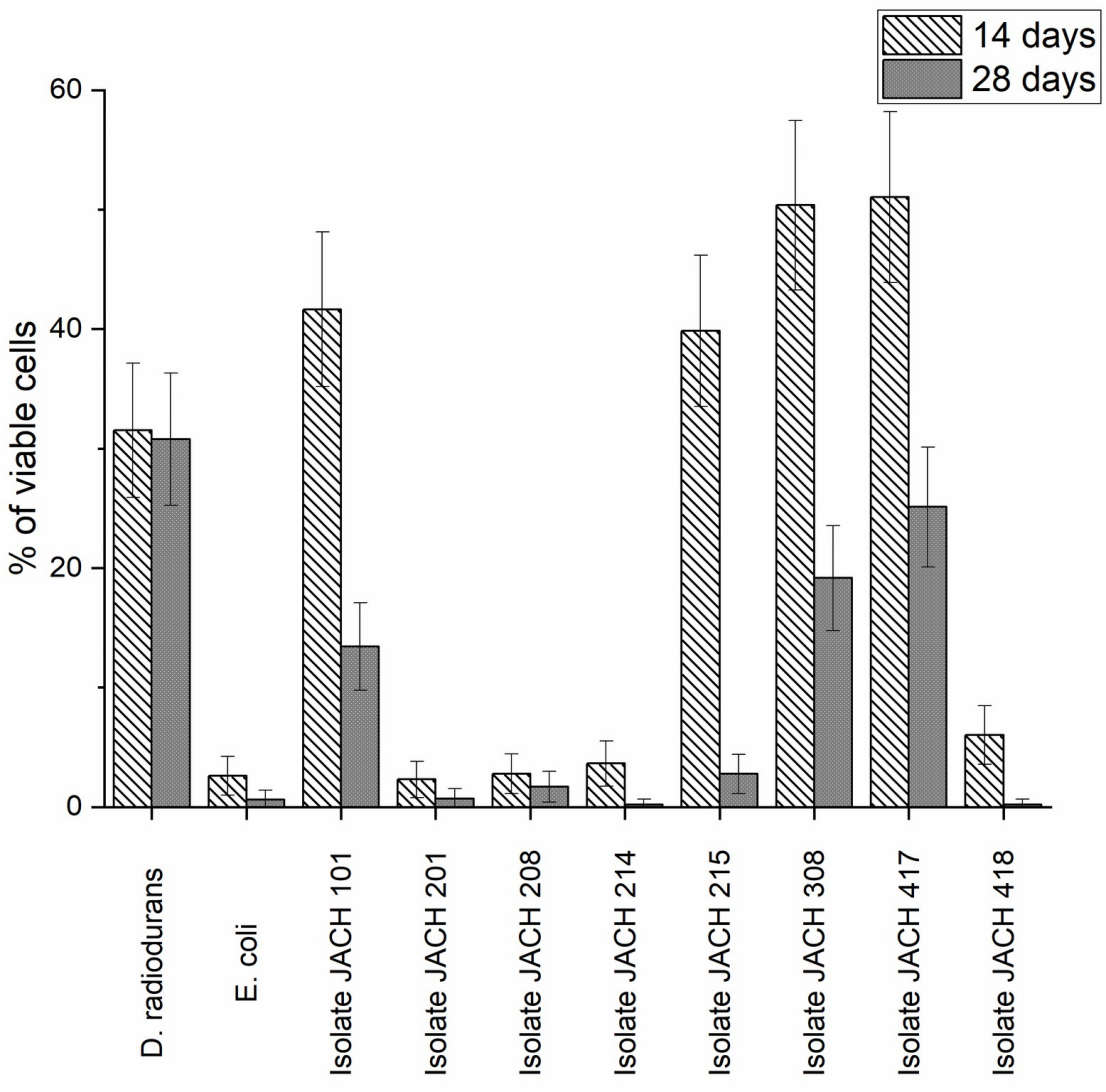
Survival of *D*. *radiodurans* R1, *E*. *coli* CCM 4517 and environmental isolates exposed to desiccation stress (viability measured after 14 days and 28 days) analysed by flow cytometry with SYTO 9 and PI.

Another factor that limits the growth of microorganisms is desiccation, which leads to cell damage similar to that caused by exposure to ionizing radiation [73]. Desiccation resistance was tested at two time points: 14 and 28 days (Fig 5). The positive control, *D*. *radiodurans* R1, exhibits the highest desiccation resistance among all tested strains, with approximately 33% of cells remaining viable after 14 days and 31% after 28 days of desiccation. On the other hand, *E*. *coli* CCM 4517 (negative control) is highly susceptible to desiccation, with survivability rate 2.6% after 14 days. Among the environmental isolates, isolates 417 (*M*. *luteus*) and 101 (*K*. *rhizophila*) not only exhibited notable resistance to radiation but also displayed robust desiccation resistance. Their resistance to desiccation surpassed even that of the positive control, *D*. *radiodurans* R1, as illustrated in Fig 5. Pathways for desiccation and starvation resistance, such as genes for osmotic stress response (*mtrB-mtrA*, *rpoE*) [74], and phosphate starvation response (*senX3-regX3*) [75] were detected in *Micrococcus* sp. KBS0714 [76]. It is assumed that the adaptation of cells to ionizing radiation evolved from desiccation tolerance [51]. This combination of extreme resistances qualifies these isolates for the classification of polyextremophiles, organisms thriving under multiple harsh conditions. This trait of multiresistance among members of the *Kocuria* sp. genus has been documented in several studies. Guesmi et al. (2021) have reported that *K*. *rhizophila* PT10 displays resilience against gamma radiation, desiccation, and hydrogen peroxide concentrations of 1% to 3% (v/v) [72]. Isolate 308 (*R*. *erythropolis/N*. *coeliaca*) and 215 (*G*. *terrae*) show relatively higher desiccation resistance after 14 days of desiccation, nonetheless they show rapid decrease in viability rates after 28 days of desiccation. The desiccation tolerance has been previously observed in *Rhodococcus* sp., such as strains *Rhodococcus* sp. A5 [77] and *Rhodococcus* sp. A27 [78]. Isolate 201 (*K*. *rhizophila*) appears to be the least resistant, followed by isolate 208 (*R*. *kristinae*), isolate 214 (*R*. *kristinae*) and 418 (*D*. *natronolimnaea*).

### 3.5 Oxidative stress tolerance

**Fig 6:**
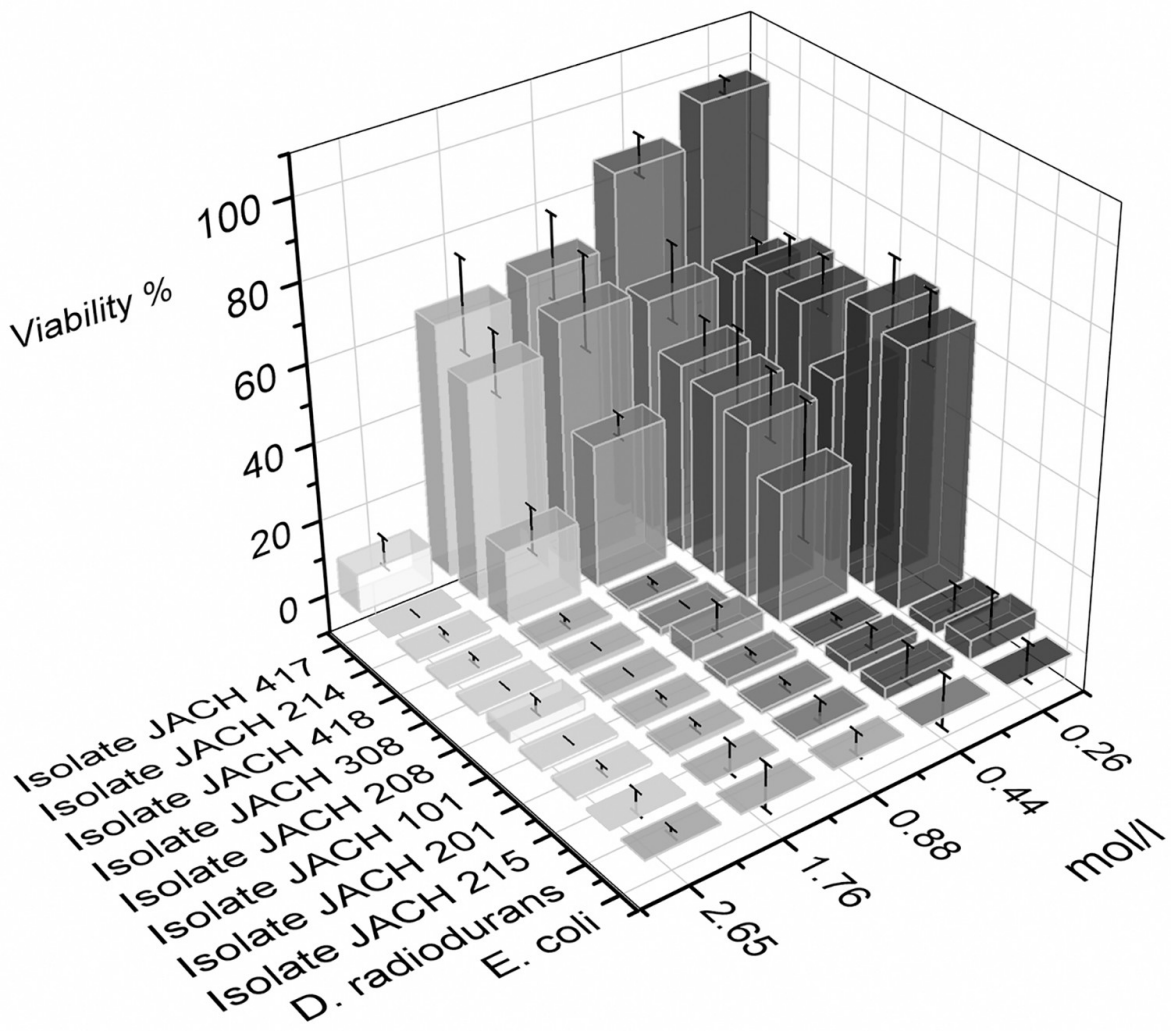
Survival of *D*. *radiodurans* R1, *E*. *coli* CCM 4517 and environmental isolates exposed to presence of H_2_O_2_ for 10 minutes.

Cell resistance to hydrogen peroxide implies an adeptness in scavenging ROS and mending oxidative stress-induced impairments [51]. The effects of varying H_2_O_2_ concentrations on bacterial isolates were investigated, and the results are presented in Fig 6. The data revealed distinct responses among the tested isolates. Isolate 214 displayed high resistance to H_2_O_2_, showing viability values of 66 ± 8% at H_2_O_2_ concentration of 1.76 mol/L. Similarly, isolate 417 (*M*. *luteus*) exhibited higher tolerance with viability values of 65 ± 2% within the same H_2_O_2_ concentration. The data obtained reveal the capability of isolates 417 (*M*. *luteus*) and 214 to withstand brief exposure to elevated concentrations of hydrogen peroxide (up to 20% v/v). This aligns with findings by Gtari et al. (2012), who observed analogous outcomes in other Actinomycetia, *Geodermatophilus obscurus* DSM 43160 and *Modestobacter multiseptatus* BC501, surviving a lethal dose of 30% (v/v) hydrogen peroxide within a 10-minute exposure timeframe [40]. In contrast, isolate 418 (*D*. *natronolimnaea*) exhibited weaker tolerance with viability values of 19 ± 4% at H_2_O_2_ concentration of 1.76 mol/L.

Conversely, isolate 101 (*K*. *rhizophila*) exhibited a notable decline in viability as H_2_O_2_ concentration increased, with viability dropping from 70 ± 9% to 1% as H_2_O_2_ concentration increased from 0.26 to 1.76 mol/L. Etemadifar et al. (2016) reported the endurance of *K*. *flava* MG5 to 4% (v/v) hydrogen peroxide, which corresponds to 1.17 mol/L [79]. Isolate 308 (*R*. *erythropolis/N*. *coeliaca*), isolate 208 (*R*. *kristinae*), and isolate 201 (*K*. *rhizophila*) all showed decreasing viability with higher H_2_O_2_ concentrations, with isolate 308 (*R*. *erythropolis/N*. *coeliaca*) and isolate 208 (*R*. *kristinae*) reaching complete loss of viability at the highest concentrations. Isolate 215 (*G*. *terrae*) demonstrated consistently low viability across all H_2_O_2_ concentrations, with viability values being close to 0.

### 3.6 Tolerance to mitomycin C

**Fig 7:**
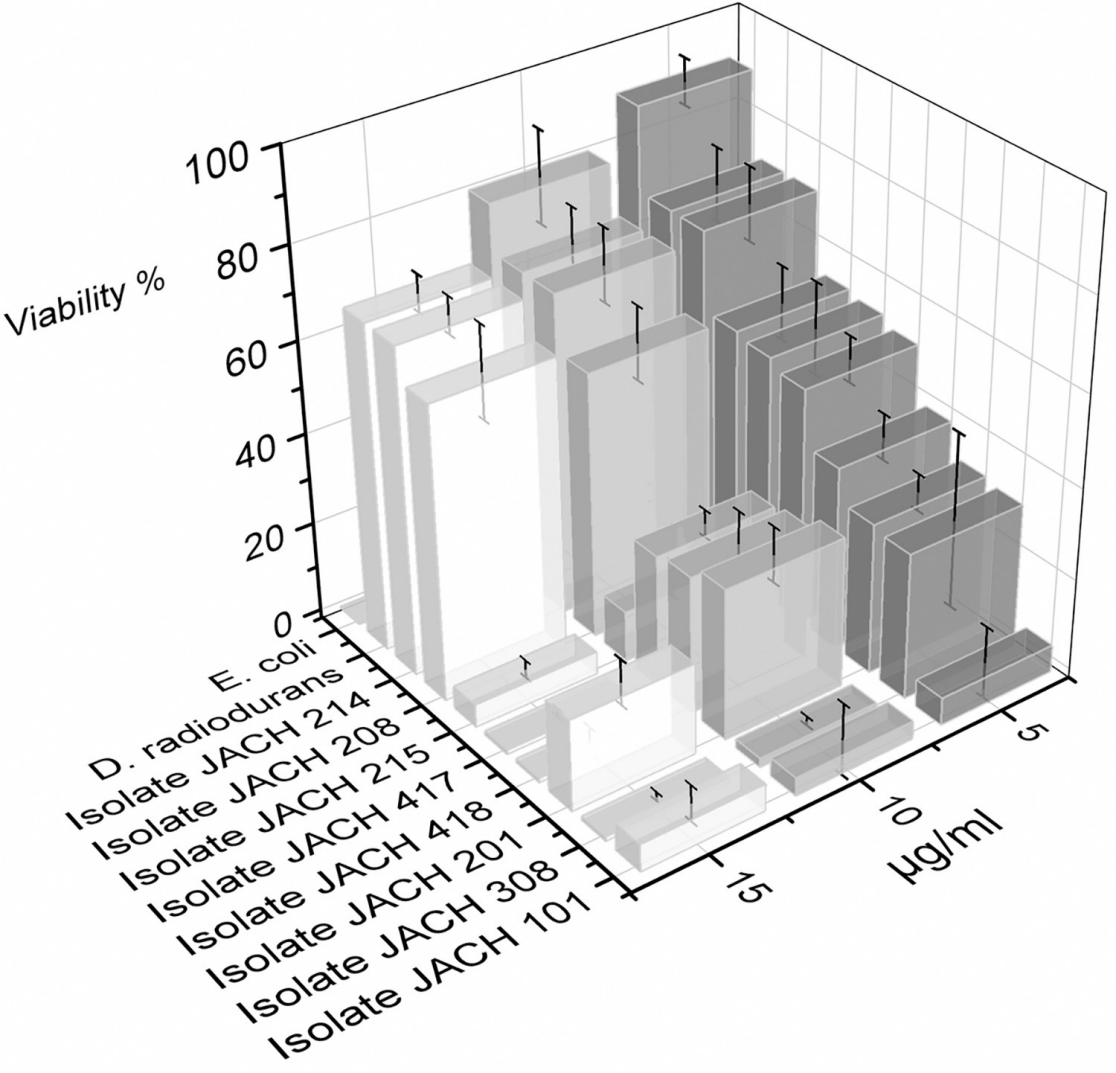
Survival of *D*. *radiodurans* R1, *E*. *coli* CCM 4517 and environmental isolates exposed to presence of mitomycin C for 30 minutes.

Mitomycin C tolerance tests are classified as tests for the resistance of microorganisms to oxidative stress [80, 81]. Mitomycin C is a genotoxic agent that induces DNA cross-links, DNA alkylation, and the production of reactive oxygen species, in the presence of O_2_, results in the generation of H_2_O_2_ [80]. Mitomycin causes inhibition of DNA synthesis, increased mutagenesis, chromosome breakage, stimulation of genetic recombination, and sister chromatid exchange [81]. The impact of varying mitomycin C concentrations on bacterial isolates was investigated and the results are summarized in Fig 7. Isolate 214 displayed consistent viability levels across the different mitomycin C concentrations, with viability of 71 ± 4% at concentration of 15 μg/mL, similarly to *D*. *radiodurans* R1. Isolate 208 also demonstrated high resistance with viability of 64 ± 10% at 15 μg/mL concentration. Most isolates displayed a significant decrease in viability as the mitomycin C concentration increased, from 54 ± 10% at 5 μg/mL to 5 ± 2% at 15 μg/mL, 33 ± 4% to 20 ± 5% for isolates 215 (*G*. *terrae*) and 201 (*K*. *rhizophila*), respectively. Some isolates reached 0% viability at the maximum concentration of mitomycin C, for example isolate 308 (*R*. *erythropolis/N*. *coeliaca*), isolate 417 (*M*. *luteus*) and isolate 418 (*D*. *natronolimnaea*). Isolate 101 (*K*. *rhizophila*) demonstrated relatively low viabilities across the mitomycin C concentrations, with values of as low as 4 ± 1%.

Both isolates 208 (*R*. *kristinae*) and 214 (*R*. *kristinae*) demonstrated resilience to mitomycin C, maintaining over 50% viability even after 60 minutes of exposure to 15 μg/mL concentration. This resistance level was on par with *D*. *radiodurans* R1. For instance, *Dietzia* MG4, exhibited survival in the presence of 8 μg/mL mitomycin C [59], which was confirmed in our study with isolate 418 (*D*. *natronolimnaea*) remaining viability after 10 μg/mL mitomycin C treatment. Based on these findings, it can be hypotheses that isolate 214 harbors proficient system for reactive oxygen species (ROS) detoxification and oxidative stress mitigation.

### 3.7 Determination of the intracellular concentrations of Mn: Fe

**Fig 8:**
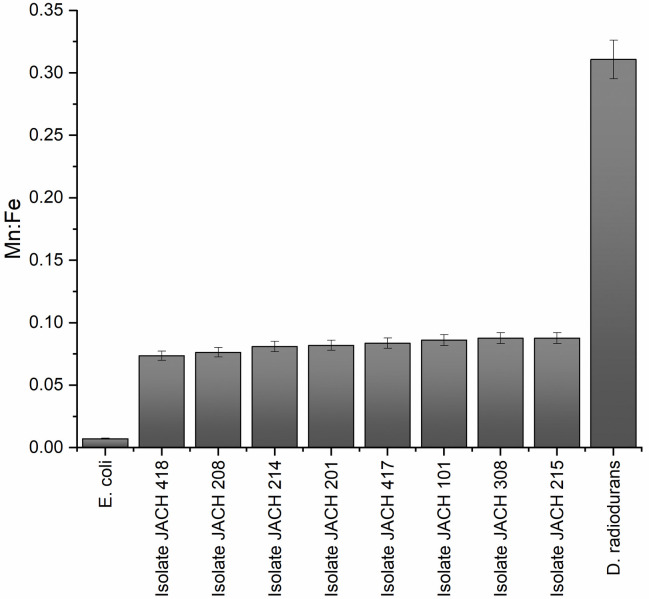
Mn:Fe ratio of *D*. *radiodurans* R1, *E*. *coli* CCM 4517 and environmental isolates studied by ICP-MS.

Investigation of intracellular Mn:Fe ratios revealed values from 0.0736 in isolate 418 (*D*. *natronolimnaea*) to 0.0878 in isolate 215 (*G*. *terrae*). Compared to control microorganisms, all studied isolates show a 10-fold higher Mn: Fe ratio than *E*. *coli* CCM 4517 (0.0072) and a 10-fold lower ration than *D*. *radiodurans* R1 (0.3108), see Fig 8.

The highest ratios were identified in isolates 215 (*G*. *terrae*), 308, 101 (*K*. *rhizophila*) and 417 (*M*. *luteus*), aligning with their increased tolerance to desiccation and, in some cases, resistance to UV-C or gamma irradiation. These results may highlight a potential link between intracellular Mn:Fe ratios, resistance to desiccation and irradiation, and between the adaptive traits exhibited by these specific bacterial isolates [55, 82, 83]. Fenton’s reaction occurs in the presence of free ferrous cations, where hydrogen peroxide undergoes decomposition, yielding a toxic hydroxyl radical. Intriguingly, unlike ferrous cations, Mn^2+^ ions remain uninvolved in this reaction. Furthermore, these manganese ions protect enzymes that house Fe-S clusters, thereby avoiding their inactivation by superoxide radicals [55, 84].

### 3.8 Isolation of lipids and fatty acid methyl ester (fame) characterization

Several isolates showed remarkable concentrations of total lipids in their dry matter. Among them, the most substantial concentration was observed in isolate 214 (*R*. *kristinae*) at 54.8% of the dry weight of the cell (CDW). Closely followed by isolate 418 (*D*. *natronolimnaea*) at 34.4% CDW and isolate 208 (*R*. *kristinae*) at 33.5% CDW. Such a high concentrations of total lipids are more common in oleogenic yeasts under -N or -N-P stress conditions [85].

The FA analysis results (S2 Table) revealed a strong similarity in the representation of most FAs among isolates that were also closer in the phylogenetic tree (Fig 2). In the first kinship cluster, isolates 101 (*K*. *rhizophila*) and 201 (*K*. *rhizophila*) both have two major FAs, palmitic (50% and 44%) and palmitoleic (47% and 40%) acids. Isolates 208 (*R*. *kristinae*) and 214 (*R*. *kristinae*) also have two major FAs, anteiso-15:0 (43% and 33%) and anteiso-17:0 (32% and 36%). Isolate 417 (*M*. *luteus*) is the least phylogenetically related in this first cluster of investigated isolates and this corresponds to the representation of FA. It has one major FA, anteiso-15:0, (70%), and three other abundant FAs, iso-15:0 (13%), iso-16:0 (7%), and anteiso-17:0 (5%). Palmitoleic acid was abundant in isolates identified as *Kocuria* sp. but is usually absent in type strains (40% for both isolates versus 0% for type strain). Similar concentrations of palmitoleic acid were found in *D*. *radiodurans*, where the content reaches 52.9% [43]. High concentrations of palmitoleic acid are more common in yeasts [85, 86]. The second cluster of phylogenetic relatedness includes 3 isolates, i.e., 215 (*G*. *terrae*), 308 (*R*. *erythropolis/N*. *coeliaca*), and 418 (*D*. *natronolimnaea*). These isolates have a majority representation of three FAs: palmitic acid (28%, 29% and 29%, respectively), further unsaturated oleic acid (14%, 35% and 5%, respectively) and finally tuberculostearic acid, represented in amounts of 44%, 28% and 45% respectively.

Comparison of the FA composition results for studied strains and respective type strains shows that the first kinship cluster differs significantly in the representation of FA compared to the type strains. These differences can indicate unique adaptive strategies and physiological characteristics among isolates, providing valuable information about their potential ecological niches and functional capabilities. In isolates 101 (*K*. *rhizophila*) and 201 (*K*. *rhizophila*), the content of unsaturated FA reaches 47% and 40% of total fatty acids respectively, in contrast to the type strain, which does not have a significant content of unsaturated FA. These isolates do not contain branched chain FAs, such as anteiso-15:0 (almost 50% in the type strain) and are replaced by straight-chain FA, i.e. palmitic acid. A similar effect also applies to anteiso-17:0, where the content increased compared to the strain type (21%). Isolates 208 (*R*. *kristinae*) and 214 (*R*. *kristinae*) also differ significantly from the type strain. The content of anteiso-15:0 decreased by half (70% in the type strain) and the content of anteiso-17:0 increased to 35% (9% in the type strain). The last isolate in the first phylogenetic cluster, which is the least related to the previous ones, isolate 417 (*M*. *luteus*), shows a similar type of differences. The content of iso-15:0 was reduced by half (26% in the type strain) and, conversely, the content of anteiso-15:0 increased by 25% (56% in the type strain).

The second related cluster in the phylogenetic tree, consisting of three isolates, has similar trends in FA changes with the type strains. In isolate 418 (*D*. *natronolimnaea*) a notable reduction was observed in the levels of unsaturated fatty acids (FAs). Particularly, the presence of palmitoleic acid, which constituted 33% in the type strain, was nearly absent in this strain. The palmitic acid content increased by 50% (14% in the type strain) as well as the tuberculostearic acid content by almost 50% (30% in the type strain). Isolate 215 (*G*. *terrae*) also lacked palmitoleic acid (16% in the type strain), but showed increased content of oleic acid by 35% (from 26% in the type strain) and by more than 60% tuberculostearic acid content (from 17% in the type strain). The isolate 308 (*N*. *coeliaca/R*. *erythropolis*) differed significantly only in the content of tuberculostearic acid, where its content increased compared to the type strain. Environmental isolates from the Mycobacteriales order, including species such as *Dietzia* sp., *Rhodococcus* sp. and *Gordonia* sp., show an increased content of tuberculostearic acid compared to the type strains. This observation suggests a potential correlation between elevated levels of tuberculostearic acid and the environmental adaptation of these isolates to the spring environment. *Mycobacteria* sp., also members of the class Actinomycetes, are widely studied for causing tuberculosis. Presence of tuberculostearic fatty acid (10Me-18:0) is typical for *Mycobacteria* sp., which contributes to the unique properties of the cell wall that make mycobacteria more resistant and less targeted by antibiotics compared to other bacteria [87]. A recent study highlights the important function of tuberculostearic acid in the compartmentalization of the mycobacterial membrane. This role appears to be central in controlling the activities of the plasma membrane, a vital barrier that allows the pathogen to tolerate stressful conditions and persist in environment [88].

The difference in FA content in type strains of *Deinococcus* and non-type strains of the same bacterium was previously described [89]. For example, the type strain *D*. *proteolyticus* isolated from the feces of *Lama glama* (Czech Collection of Microorganisms CCM 2703) differs from *D*. *proteolyticus* (Japan Atomic Research Institute) in the content of some FAs, namely 16:0, i.e. 2.9% in the type strain versus 21.1% of total FAs in the strain from Atomic Institute. Also, in two strains of *Kocuria rhizophila* (JACH 101 and JACH 201) compared to the type strain [90] a decrease of 16:0 in the type strain (2.6%) versus 49.5 and 43.8% of springs in Jáchymov. Conversely, i17:0 FA is absent in the type strain but constitutes 21.5% of total FA in the strain from the Atomic Institute. Likewise, i17:0 FA represents 1.2% of total FA in the type strain, whereas strains from Jáchymov springs contain significantly lower levels of this acid, approximately one-tenth of the aforementioned quantity.

The presented data emphasizes the varied reactions of different microorganisms to environmental stress factors. For a closer understanding, it is necessary to focus on the overall lipidomic profile determination, for example, by shotgun lipidomic analysis. This approach facilitates the identification of numerous molecular species of lipids, potentially numbering in the thousands. Through shotgun analysis, even atypical, infrequently encountered, or entirely novel lipid classes can be discerned, as exemplified in studies involving thermophilic bacteria [91, 92].

The unusually high content of lipids, including the representation of fatty acids, can be explained, for example, by the following hypothesis. The solubility of radon in lipids is two orders of magnitude higher than its solubility in water [93, 94]. In the article by Nussbaum and Hursh [93], the solubility coefficient of radon was investigated at different temperatures, mainly in fatty acids. The amount of soluble Rn in FA increases along with the chain length (decreasing solubility of FA in water) and decreases slightly with temperature. Sanjon et al. describe that radon concentration-specific solubility is approximately two orders of magnitude lower in saline solution than in oleic or linoleic acids [94]. It was also found that the difference between saturated and unsaturated lipid molecules plays only a minor role in the concentration-specific solubility of radon. Again, this finding explains the high representation of both palmitic and palmitoleic acids because, from the point of view of the cell, the unsaturation of the hydrocarbon chain is negligible. Based on these published experimental data, it is possible to assume that bacteria living in radioactive water with their high lipid content, especially in their cell walls, absorb Rn, thereby preventing its further penetration into the cell interior, which could damage DNA due to radioactive decay. This is another possible mechanism by which bacteria can cope with radioactive radiation.

## 4 Conclusion

In this study, we have isolated and thoroughly characterized environmental bacterial strains from the adverse conditions of radon springs. Extremophilic microorganisms often serve as sources of industrially important enzymes and other compounds. Therefore, our objective was to assess the resistance of these isolates to various extreme conditions, potentially enhancing their biotechnological applications. Numerous studies confirm the multiresistance potential of various members of Actinomycetia class, which proved to be abundant in the environment of the radon springs. We have further confirmed that several strains are indeed multiresistant to oxidative stress and irradiation.

The isolates 101 and 201 can be classified as radioresistant based on their resistance to UV-C and γ-radiation. These isolates also show high content of unsaturated fatty acids (47 and 40%, respectively). In comparison with *Deinococcus* sp., the isolates 101 and 201 had a similar fatty acids composition [95–97]. Isolate 417 showed the broadest resistance profile being moderately resistant to UV-C, highly resistant to gamma irradiation, H_2_O_2_ and desiccation. Resistance to desiccation was further confirmed for *Rhodococcus* sp. with isolate 308 remaining high viability after long-term desiccation.

Another interesting group of multiresistant isolates were isolates 208 and 214, which had high resistance to the effects of mitomycin, comparable to *D*. *radiodurans*. In addition, isolate 214 was highly resistant to the effects of H_2_O_2_ and also had the highest total lipid content, nearly 55%. Interestingly, although *D*. *radiodurans* is highly resistant to the DNA-damaging agent mitomycin, it is not resistant to the oxidative action of H_2_O_2_. Isolate 214 combines both of these resistances. Although the fatty acids consist only of saturated fatty acids, due to the multiresistance of this isolate, further studies would be worth considering. It is possible that special groups of lipids that are found in other extremophilic bacteria with similar fatty acid composition will be discovered [91].

Lipidomic analysis revealed several significant differences between environmental strains and type strains. These differences might be linked to the specifics of the environment and adverse conditions of the radon springs. High Mn:Fe ration further correlate with the resistance of all environmental isolates.

In conclusion, our findings suggest that the multiresistant strains obtained in this study exhibit promising characteristics, and their resistance mechanisms require further investigation. Future studies should focus on validating these mechanisms and assessing the biotechnological potential of these unique strains, which could have valuable applications in various industrial and environmental contexts.

## Supporting information

S1 Table Biochemical analysis of isolated strains by GEN III Microplate (Biolog). (XLSX)

S2 Table Fatty acid composition of isolates from this study and the closest type strains. (DOCX)
