# Supplementary material for: Isolation and characterization of multiple-stress tolerant bacteria from radon springs
Source: PLoS One. 2024 Mar 7;19(3):e0299532. doi: 10.1371/journal.pone.0299532 (PMC10919644; doi:10.1371/journal.pone.0299532)
Supplement: S2 Table — (DOCX) [file pone.0299532.s002.docx]

Table S2. Fatty acid composition of isolates from this study and the closest type strains.

|  | JACH 101 | JACH 201 | *Kocuria rhizophila* | JACH 208 | I JACH 214 | *Rothia kristinae* | JACH 215 | *Gordonia terrae* | JACH 308 | *Rhodococcus erythropolis* | JACH 417 | *Micrococcus luteus* | JACH 418 | *Dietzia natronolimnaea* |
| --- | --- | --- | --- | --- | --- | --- | --- | --- | --- | --- | --- | --- | --- | --- |
| % CDW | 17.3 | 23.7 |  | 33.5 | 54.8 |  | 11.8 |  | 17.7 |  | 31.5 |  | 34.4 |  |
| UNSATURATED | 47.0 | 39.6 |  | 0.0 | 0.5 |  | 34.7 | 42.0 | 5.9 | 4.5 | 0.0 | 3.5 | 16.1 | 53.2 |
| SATURATED | 53.0 | 60.4 | 96.5 | 100.0 | 99.5 | 98.4 | 65.3 | 49.0 | 94.1 | 59.9 | 100.0 | 93.8 | 83.9 | 46.8 |
|  |  |  |  |  |  |  |  |  |  |  |  |  |  |  |
| C13:0 | 0.0 | 1.5 |  | 0.7 | 2.3 |  | 0.8 |  | 2.4 |  | 0.8 |  | 1.4 |  |
| i14:0 | 0.0 | 0.0 | 1.1 | 0.5 | 0.3 | 2.6 | 0.0 |  | 0.0 |  | 1.8 | 2.9 | 0.0 |  |
| C14:0 | 0.2 | 4.0 | 2.4 | 0.1 | 0.2 | 1.0 | 1.1 |  | 6.3 | 6.9 | 0.2 | 5.5 | 0.6 | 1.0 |
| i15:0 | 1.2 | 6.6 | 13.8 | 0.0 | 0.7 | 1.6 | 0.0 |  | 0.0 |  | 13.1 | 26.1 | 0.0 |  |
| a15:0 | 0.0 | 0.0 | 48.4 | 43.0 | 33.4 | 70.1 | 0.0 |  | 0.0 |  | 70.3 | 56.2 | 0.0 |  |
| C15:0 | 0.0 | 0.0 |  | 0.3 | 0.2 |  | 0.2 |  | 2.4 | 3.5 | 0.2 |  | 0.7 |  |
| i16:0 | 0.5 | 2.6 | 5.9 | 16.6 | 15.9 | 12.1 | 0.0 |  | 0.0 |  | 7.0 | 1.9 | 0.0 |  |
| 16:1w7 | 47.0 | 39.6 |  | 0.0 | 0.1 |  | 0.0 | 16.0 | 0.7 |  | 0.0 | 3.5 | 2.3 | 33.0 |
| C16:0 | 49.5 | 43.8 | 2.6 | 5.8 | 8.0 | 1.7 | 29.1 | 32.0 | 28.8 | 25.2 | 0.9 | 1.2 | 28.3 | 14.1 |
| 10Me-16:0 | 0.0 | 0.0 |  | 0.0 | 0.0 |  | 0.0 |  | 3.5 | 2.9 | 0.0 |  | 0.4 | 0.7 |
| i17:0 | 0.1 | 0.0 | 1.2 | 0.3 | 0.2 |  | 0.0 |  | 0.0 |  | 0.3 |  | 0.0 |  |
| a17:0 | 1.4 | 1.9 | 21.1 | 31.9 | 35.8 | 9.3 | 0.0 |  | 0.0 |  | 5.0 |  | 0.0 |  |
| C17:0 | 0.0 | 0.0 |  | 0.0 | 0.1 |  | 0.7 |  | 1.8 | 1.8 | 0.0 |  | 3.7 |  |
| 10Me-17:0 | 0.0 | 0.0 |  | 0.0 | 0.0 |  | 0.0 |  | 2.3 | 2.8 | 0.0 |  | 0.8 | 0.8 |
| 18:1w9 | 0.0 | 0.0 |  | 0.0 | 0.2 |  | 34.7 | 26.0 | 5.2 | 4.5 | 0.0 |  | 13.6 | 15.7 |
| 18:1w7 | 0.0 | 0.0 |  | 0.0 | 0.2 |  | 0.0 |  | 0.0 |  | 0.0 |  | 0.2 | 4.5 |
| C18:0 | 0.1 | 0.0 |  | 0.8 | 2.4 |  | 5.5 |  | 1.8 | 0.8 | 0.4 |  | 3.7 |  |
| 10Me-18:0 | 0.0 | 0.0 |  | 0.0 | 0.0 |  | 27.9 | 17.0 | 44.8 | 16.0 | 0.0 |  | 44.3 | 30.2 |
| SOURCE | This study | This study | (1) | This study | This study | (2) | This study | (3) | This study | (4) | This study | (2) | This study | (5) |

1. Kovacs G, Burghardt J, Pradella S, Schumann P, Stackebrandt E, Marialigeti K. Kocuria palustris sp. nov. and Kocuria rhizophila sp. nov., isolated from the rhizoplane of the narrow-leaved cattail (Typha angustifolia). International Journal of Systematic Bacteriology. 1999;49:167-73.

2. Stackebrandt E, Koch C, Gvozdiak O, Schumann P. TAXONOMIC DISSECTION OF THE GENUS MICROCOCCUS - KOCURIA GEN-NOV, NESTERENKONIA GEN-NOV, KYTOCOCCUS GEN-NOV, DERMACOCCUS GEN-NOV, AND MICROCOCCUS COHN 1872 GEN EMEND. International Journal of Systematic Bacteriology. 1995;45(4):682-92.

3. Goodfellow M, Kumar Y, Maldonado LA. Gordonia. Bergey's Manual of Systematics of Archaea and Bacteria. p. 1-29.

4. Jones AL, Goodfellow M. Rhodococcus. Bergey's Manual of Systematics of Archaea and Bacteria. p. 1-50.

5. Gharibzahedi SMT, Razavi SH, Mousavi SM. Characterization of bacteria of the genus Dietzia: an updated review. Annals of Microbiology. 2014;64(1):1-11.
